# Identification of Inherited Retinal Disease-Associated Genetic Variants in 11 Candidate Genes

**DOI:** 10.3390/genes9010021

**Published:** 2018-01-10

**Authors:** Galuh D. N. Astuti, L. Ingeborgh van den Born, M. Imran Khan, Christian P. Hamel, Béatrice Bocquet, Gaël Manes, Mathieu Quinodoz, Manir Ali, Carmel Toomes, Martin McKibbin, Mohammed E. El-Asrag, Lonneke Haer-Wigman, Chris F. Inglehearn, Graeme C. M. Black, Carel B. Hoyng, Frans P. M. Cremers, Susanne Roosing

**Affiliations:** 1 Department of Human Genetics, Radboud University Medical Center, 6525 GA Nijmegen, The Netherlands; Galuh.Astuti@radboudumc.nl (G.D.N.A.); MuhammadImran.Khan@radboudumc.nl (M.I.K.); Lonneke.Haer-Wigman@radboudumc.nl (L.H.-W.); Frans.Cremers@radboudumc.nl (F.P.M.C.); 2 Radboud Institute for Molecular Life Sciences, Radboud University, 6525 GA Nijmegen, The Netherlands; 3 The Rotterdam Eye Hospital, 3011 BH Rotterdam, The Netherlands; Born@eyehospital.nl; 4 Donders Institute for Brain, Cognition and Behaviour, Radboud University Nijmegen, 6525 EN Nijmegen, The Netherlands; 5 Institut National de la Santé et de la Recherche Médicale, Institute for Neurosciences of Montpellier, 34080 Montpellier, France; Beatrice.Bocquet@inserm.fr (B.B.); Gael.Manes@inserm.fr (G.M.); 6 University of Montpellier, 34090 Montpellier, France; 7 CHRU, Genetics of Sensory Diseases, 34295 Montpellier, France; 8 Department of Computational Biology, Unit of Medical Genetics, University of Lausanne, 1015 Lausanne, Switzerland; Mathieu.Quinodoz@unil.ch; 9 Section of Ophthalmology & Neuroscience, Leeds Institute of Biomedical & Clinical Sciences, University of Leeds, St. James’s University Hospital, LS9 7TF Leeds, UK; M.Ali@leeds.ac.uk (M.A.); C.Toomes@leeds.ac.uk (C.T.); M.E.Elasrag@leeds.ac.uk (M.E.E.-A.); C.Inglehearn@leeds.ac.uk (C.F.I.); 10 Department of Ophthalmology, St. James’s University Hospital, LS9 7TF Leeds, UK; Martin.McKibbin@nhs.net; 11 Department of Zoology, Faculty of Science, Benha University, 13511 Benha, Egypt; 12 Centre for Genomic Medicine, St. Mary’s Hospital, Manchester Academic Health Science Centre, University of Manchester, M13 9PL Manchester, UK; Graeme.Black@manchester.ac.uk; 13 Department of Ophthalmology, Radboud University Medical Center, 6525 EX Nijmegen, The Netherlands; Carel.Hoyng@radboudumc.nl

**Keywords:** whole exome sequencing, inherited retinal diseases, candidate retinal disease genes

## Abstract

Inherited retinal diseases (IRDs) display an enormous genetic heterogeneity. Whole exome sequencing (WES) recently identified genes that were mutated in a small proportion of IRD cases. Consequently, finding a second case or family carrying pathogenic variants in the same candidate gene often is challenging. In this study, we searched for novel candidate IRD gene-associated variants in isolated IRD families, assessed their causality, and searched for novel genotype-phenotype correlations. Whole exome sequencing was performed in 11 probands affected with IRDs. Homozygosity mapping data was available for five cases. Variants with minor allele frequencies ≤ 0.5% in public databases were selected as candidate disease-causing variants. These variants were ranked based on their: (a) presence in a gene that was previously implicated in IRD; (b) minor allele frequency in the Exome Aggregation Consortium database (ExAC); (c) in silico pathogenicity assessment using the combined annotation dependent depletion (CADD) score; and (d) interaction of the corresponding protein with known IRD-associated proteins. Twelve unique variants were found in 11 different genes in 11 IRD probands. Novel autosomal recessive and dominant inheritance patterns were found for variants in Small Nuclear Ribonucleoprotein U5 Subunit 200 (*SNRNP200*) and Zinc Finger Protein 513 (*ZNF513*), respectively. Using our pathogenicity assessment, a variant in DEAH-Box Helicase 32 (*DHX32*) was the top ranked novel candidate gene to be associated with IRDs, followed by eight medium and lower ranked candidate genes. The identification of candidate disease-associated sequence variants in 11 single families underscores the notion that the previously identified IRD-associated genes collectively carry > 90% of the defects implicated in IRDs. To identify multiple patients or families with variants in the same gene and thereby provide extra proof for pathogenicity, worldwide data sharing is needed.

## 1. Introduction

Inherited retinal diseases (IRDs) comprise of a clinically and genetically heterogeneous group of hereditary retinal degenerations, which affect around 1 in 3000 people. Inherited Retinal Diseases may occur both in syndromic and non-syndromic forms, and can be inherited in all the Mendelian inheritance patterns. Inherited Retinal Diseases are one of the leading causes of vision loss in young individuals in the developed countries, with a significant impact on patients and society [1,2,3]. In the past two decades, new therapies have been developed that rely on knowledge of the underlying genetic defects [4,5,6].

The high genetic diversity and overlapping phenotypes of IRDs pose significant challenges in the genetic elucidation of these disorders. The spectrum of genotyping technologies employed in the last three decades, ranging from positional cloning, (mouse) candidate disease gene analyses, linkage analysis, homozygosity mapping, and whole exome sequencing (WES) have led to a wide body of knowledge regarding the molecular genetics of IRDs. Currently, mutations in 261 genes have been discovered to be involved in IRDs [7]. Nevertheless, there is a trend in which the more recently discovered IRD-associated genes are responsible for a very small percentage of cases, in comparison to the genes that were discovered in the earlier studies. Two WES studies in the Saudi-Arabian and the Dutch population showed that only ~3–4% of IRD cases carry variants in novel IRD-associated genes [8,9,10,11,12]. Consequently, finding additional cases carrying variant(s) in the same candidate gene is often challenging.

A large cohort of IRD cases is crucial to assess the contribution of unique variants in causing diseases. The European Retinal Diseases Consortium (ERDC) [13], consisting of 17 research groups, was formed as a collaborative effort to elucidate molecular genetics causes of IRDs. Within the ERDC, information on candidate IRD genes was shared which enabled the identification of multiple cases or families with variants in the same gene [14,15,16,17].

In this study, we report on the identification of potential candidate disease variants in 11 genes that were found in single families. We assessed the causality of these candidate disease variants, searched for novel genotype-phenotype correlations and investigated the occurrence of different inheritance patterns for variants in known IRD-associated genes.

## 2. Materials and Methods

### 2.1. Subject and Clinical Evaluation

**Figure 1:**
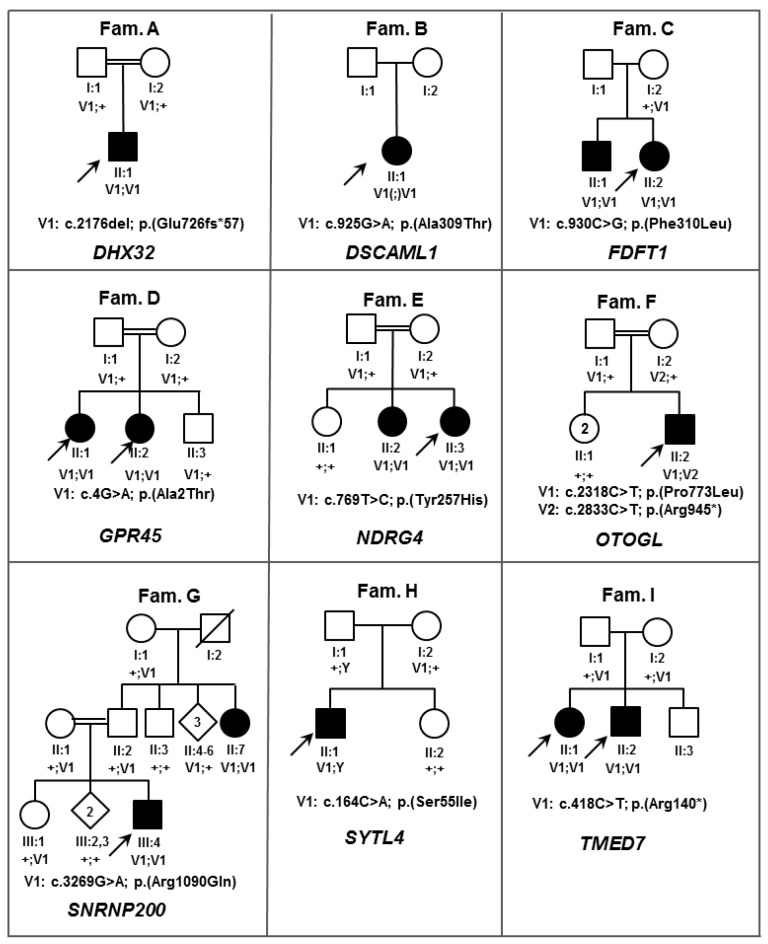
Pedigrees of families (Fam) harboring inherited retinal diseases (IRD) candidate gene variants inherited in an autosomal recessive manner. Double lines denote consanguinity, open squares and circles represent healthy males and females respectively, black squares and circles represent affected males and females respectively, diagonal line indicates deceased person, arrows point to the probands, Roman numbers denote the generation number, Greek numbers address the individual in each generation, V1 and V2 depicts the variants in the families, Y denotes Y-chromosome, ‘+’ indicates a wild type allele, (;) indicates that segregation analysis was not performed. *DHX32*: DEAH-Box Helicase 32; *DSCAML1*: DS Cell Adhesion Molecule Like 1; *FDFT1*: Farnesyl-Diphosphate Farnesyltransferase 1; *GPR45*: G Protein-Coupled Receptor 45; *NDRG4*: NDRG Family Member 4; *OTOGL*: Otogelin Like; *SNRNP200*: Small Nuclear Ribonucleoprotein U5 Subunit 200; *SYTL4*: Synaptotagmin Like 4; *TMED7*: Transmembrane P24 Trafficking Protein 7.

**Figure 2:**
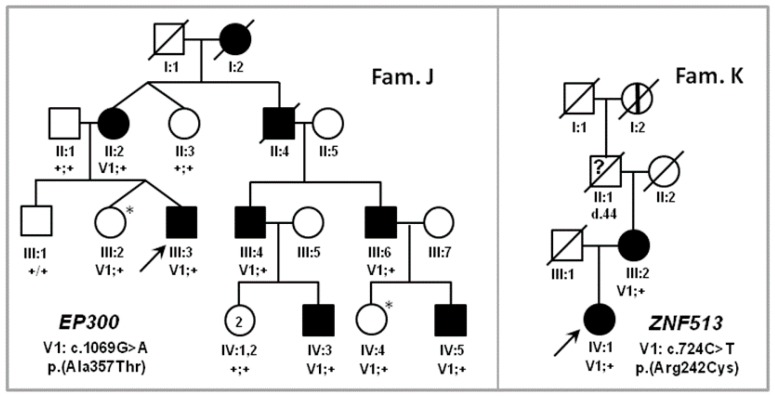
Pedigrees of families harboring IRD candidate gene variants inherited in an autosomal dominant manner. Open squares and circles represent healthy males and females, black squares and circles represent affected males and females, and diagonal line indicates deceased person, arrows point to the probands. The question mark depicts a person who deceased too young to possibly develop the disease. The vertical line represents a person affected by hearsay. Asterisks (*) denote non-penetrant persons. Roman numbers denote the generation number, Greek numbers address the individual within in the generation, V1 depicts the variant in the families, ‘+’ indicates a wild-type allele. *EP300*: E1A Binding Protein P300; *ZNF513*: Zinc Finger Protein 513.

The study protocol adhered to the tenets of the declaration of Helsinki and received approval from the respective local ethics committees (2011, MEC-2010-359, ABR-NL34152.078.10; 2004, 03/362 17/YH/0365; 2015, 176953, 15/YH/0365; 2008-A01238-47). Written informed consent was obtained from all participants or parents of children prior to their inclusion in this study. The 11 probands belong to eight autosomal recessive retinitis pigmentosa (RP) families, two autosomal dominant RP families, one autosomal recessive cone-rod dystrophy (CRD) family, one achromatopsia (Figure 1 and Figure 2, Table 1 and Table 2). Each patient was reviewed for clinical examination including visual acuity, fundus photography, fundus autofluorescent, and optical coherence tomography.

### 2.2. Genetic Analysis

Genomic DNA was isolated from peripheral blood lymphocytes according to standard salting out procedures. Whole exome sequencing analysis consisting of targeted capture, sequencing platform, and mapping was independently performed by each center using several types of software (Supplementary Table S1) [18,19,20]. Sequences were aligned to National Center for Biotechnology Information (NCBI) Build 37 of the human genome [21] using corresponding mapping tools and copy number variant analyses were performed. Single nucleotide polymorphism (SNP) microarray analysis was performed in five families and the runs of homozygosity were assessed using PLINK or Homozygosity mapper [20,22].

### 2.3. Variant Prioritization

Whole exome sequencing identified approximately 120,000 variants in each proband. Inclusion of rare variants with minor allele frequency (MAF) ≤ 0.5% was performed as an initial filtering step. Thereafter, protein coding and splice site variants were filtered and assessed using in silico prediction tools to prioritize the remaining variants. Four parameters were used to assess the pathogenicity of candidate variants: (a) minor allele frequencies obtained from the Exome Aggregation Consortium (ExAC) [23] database containing 60,706 exomes of unrelated healthy individuals, accessed September 2017; (b) in silico pathogenicity assessment using the combined annotation dependent depletion (CADD) score; (c) interaction with known IRD-associated proteins; and (d) known association of the relevant gene with IRDs. Using these criteria, all of the candidate genes were scored and ranked according to the number of matching parameters. The total score corresponded to the likelihood of a gene to be associated with IRDs. Yet Another Scientific Artificial Reality Application (Yasara) [24] was used to model the possible three-dimensional (3D) structural alterations caused by Small Nuclear Ribonucleoprotein U5 Subunit 200 (SNRNP200) amino acid changes [25]. Primers for candidate variants confirmation were designed with Primer3 plus [26]. 

### 2.4. Data Sharing

Each ERDC partner shared candidate genes in biannual meetings. Thereafter, the corresponding genes were screened for the presence of variants in WES data and homozygosity mapping data were assessed for homozygous regions larger than 4 Mb containing these candidate genes. In addition, candidate genes were posted at the open access ERDC website to increase the probability of finding additional cases carrying variants in the same candidate genes [13]. 

## 3. Results

**Figure 3:**
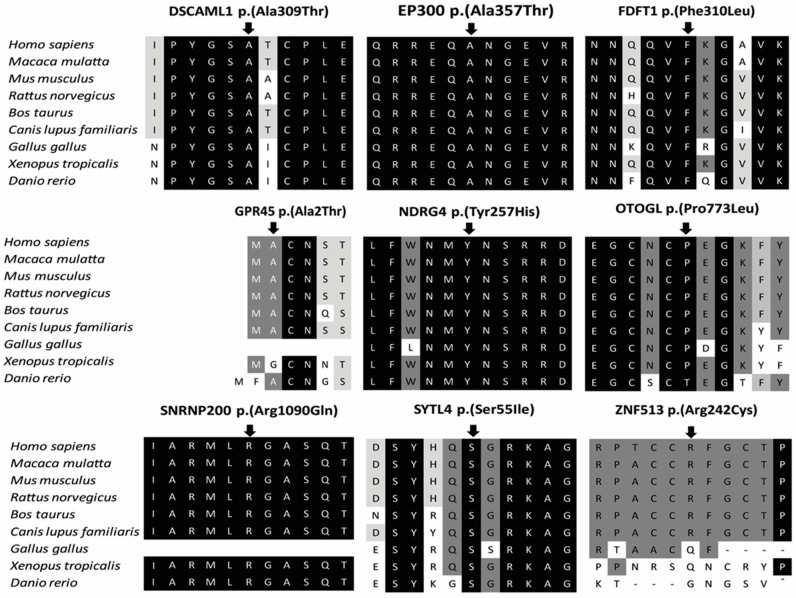
Amino acid conservation for the missense variants identified in this study. DSCAML1: DS Cell Adhesion Molecule Like 1; EP300: E1A Binding Protein P300; FDFT1: Farnesyl-Diphosphate Farnesyltransferase 1; GPR45: G Protein-Coupled Receptor 45; NDRG4: NDRG Family Member 4; OTOGL: Otogelin Like; SNRNP200: Small Nuclear Ribonucleoprotein U5 Subunit 200; SYTL4: Synaptotagmin Like 4; ZNF513: Zinc Finger Protein 513.

We performed an assessment to predict the pathogenicity of selected candidate variants by combining: (a) minor allele frequency (ExAC); (b) in silico prediction program (CADD score); (c) whether there is any interaction with retinal-associated protein (from previous studies or STRING protein network database) [27]; and, (d) whether there is any described association with IRDs. Subsequently, the total score was used to consider the likelihood of variants to be causative. In addition, we also implemented the American College of Medical Genetics (ACMG) guidelines to predict variants based on their pathogenicity [28]. Upon this assessment, variants in the known IRD-associated gene *SNRNP200* is the highest ranked classification ‘score 4’. For the other genes, two genes have a ‘score 3’ using our assessment, i.e., DEAH-Box Helicase 32 (*DHX32*) and Zinc Finger Protein 513 (*ZNF513*). The eight remaining genes have a lower pathogenicity scoring with ‘score 2’ or low pathogenicity scoring of ‘score 1’. Hence, these genes are better candidate in comparison to the other genes. In addition, we provide the amino acid conservation for all the missense variants identified in this study (Figure 3). Clinical details of affected individuals of all families are described in Table 2.

### 3.1. Score 4 Candidate IRD Gene

**Figure 4:**
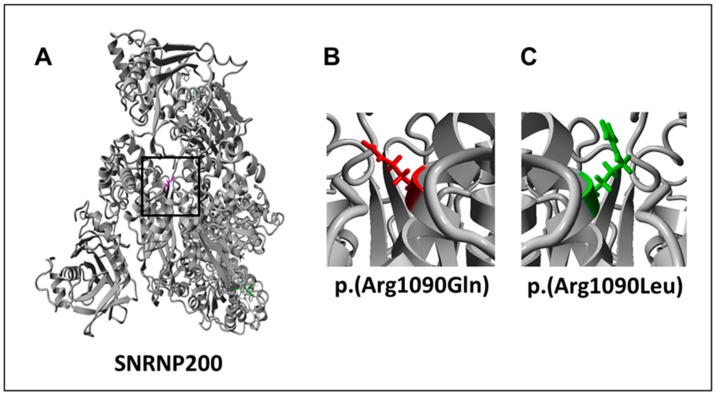
3D protein structure modeling for missense variants in SNRNP200 due to autosomal dominant and recessive variants. (**A**) An overview of the 3D protein structure model for SNRNP200. Arg1090 is located in the center of the model indicated by the square. (**B**) Modeling of the missense variant identified in family G. The autosomal recessive variant alters to glutamine that is uncharged but has the same polarity as the wild type amino acid. (**C**) The previously described SNRNP200 variant p.(Arg1090Gln) leads to a dominantly inherited retinitis pigmentosa (RP). The wild type arginine is altered to a nonpolar and hydrophobic leucine.

In the consanguineous Pakistani family G, we performed microarray SNP analysis prior to WES. In the largest homozygous region (14.6 Mb), we identified a homozygous c.3269G>A (p.(Arg1090Gln)) variant in *SNRNP200*. This variant occurs in 3 of 121,400 (0.000025) alleles in ExAC and is absent in 180 ethnically matched in-house controls. The substitution affects a highly conserved amino acid and has a PhyloP score [29] of 5.77 (range-14.1; 6.4). The new residue has a small physicochemical difference in comparison to the wild type amino acid, with a Grantham [30] score of 43 (range 0–215). The wild type arginine residue in position 1090 is located in the ratchet helix of the helical bundle (HB) of human Bad Response to Refrigeration 2 (hBRR2) that forms a unidirectional tunnel for RNA molecules to promote RNA unwinding events (Figure 4A,B). Arg1090 seems to be in direct contact with the RNA molecule and an alteration in this position may disrupt the unwinding of hBRR2.

### 3.2. Score 3 Candidate IRD Genes

The *DHX32* extension variant c.2176del (p.(Glu726Asnfs*57)) in family A will result in a frameshift with read-through of the stop codon resulting in a transcript encoding 57 additional amino acids when compared to the wild type protein. This variant has a minor allele frequency of 0.000016 in the ExAC database. DHX32 is one of DEAD (Asp-Glu-Ala-Asp) box proteins with a putative role as a RNA helicase that is involved in nuclear regulation and mitochondrial gene expression. As the variant occurs in the last exon of *DHX32* there will be no nonsense mediated decay. The addition of 57 amino acids could however destabilize the protein’s folding or it might interfere in protein-protein interactions.

The third candidate gene is *ZNF513*. The variant c.724C>T (p.(Arg242Cys)) was identified in two individuals from family K affected with late onset autosomal dominant RP. This variant is absent in the ExAC database. The variant affects a highly conserved amino acid and substitutes a nucleotide with a PhyloP score of 2.71. In addition, the wild type arginine residue has a large physicochemical difference with cysteine, with a Grantham score of 180. Based on the family history (grandfather deceased at age 44 years when disease may not yet have appeared; great-grandfather mentioned to be blind) there is strong supporting evidence for a dominant inheritance pattern. To further rule out autosomal recessive inheritance, a detailed analysis of copy number variants was performed. In the fully covered gene, there was no evidence for the presence of deletions or duplications in *ZNF513* in the affected proband.

### 3.3. Score 2 Candidate IRD Genes

In family B, we identified a homozygous missense c.925G>A (p.(Ala309Thr)) variant in *DSCAML1*, a gene that encodes the Down syndrome cell adhesion molecule-like 1 transmembrane protein. The substitution occurs in the amino acid that is highly conserved. The variant affects a highly conserved nucleotide with a PhyloP score of 5.86, but the altered amino acid is expected to have a relatively small physicochemical difference with a Grantham score of 58. This variant is found in 0.000022 of alleles in the ExAC database. All of the in silico prediction programs predicted this substitution to be deleterious, supported by the high CADD score of 34. The variant is located in the region that encodes the Immunoglobulin C2-type domain of the gene.

Down syndrome cell adhesion molecule (DSCAM) and DSCAML1 are known to be cell adhesion molecules that play roles in several neurodevelopmental functions, including neuronal self-avoidance [33]. This adhesion molecule is expressed in a subset of amacrine cells and most retinal ganglion cells (RGCs) soon after they develop and promotes laminar specificity in the retinal synaptic connections via homophilic intracellular interactions [33]. A recent study in mice shows the expression of *Dscaml1* in the rod circuit, and the lack of *Dscaml1* leads to fasciculate rod bipolar cell dendrites and clumped amacrine cell bodies, suggesting its role in the neuronal self-avoidance. Moreover, this study also suggested the importance of *Dscaml1* for rod bipolar cell dendrite organization and amacrine cell mosaic patterning [34].

Segregating compound heterozygous variants c.2318C>T and c.2833C>T (p.(Pro773Leu) and p.(Arg945*)) were identified in Otogelin Like (*OTOGL*) in family F with non-syndromic autosomal recessive RP. The missense variant alters a highly conserved nucleotide and amino acid (proline), with a conserved nucleotide with PhyloP score of 4.00. This variant occurs in 89/118,882 alleles (0.00075) in the ExAC database, i.e., heterozygously in 85 persons and homozygously in two persons. The allele frequency of the nonsense variant was 0.000076 in the ExAC database and predicted to result in nonsense mediated decay. If there would be a shortened protein, it will no longer include two von Willebrandt factor, type D domains, two C8-domains, and the C-terminal domain. Pathogenic mutations in OTOGL are associated with sensorineural hearing loss [35,36]. Based on their allele frequencies, we considered both variant to be less likely associated with the disease phenotype; however, no other outstanding variants were observed. Homozygosity mapping with the 250.000 SNP array had indicated two homozygous regions in which no currently known IRD-associated genes appear.

In the four-generation family J, we detected a heterozygous missense c.1069G>A (p.(Ala357Thr)) variant in the E1A Binding Protein P300 (*EP300*) gene that inherited in an autosomal dominant manner. The variant alters a moderately conserved amino acid and affects a highly conserved nucleotide (PhyloP: 6.34). This substitution is absent from the ExAC database and all in silico predictions show this variant to be pathogenic. It occurs in exon 4 that encodes the transcription adaptor putative Zinc finger domain of the EP300 protein. The *EP300* gene encodes p300 histone acetyltransferase that modulate transcription through chromatin remodeling and also plays role in cell proliferation and differentiation [37]. EP300 is ubiquitously expressed in adult tissue. Heterozygous truncating variants in this gene have previously been associated with the Rubinstein-Taybi syndrome, a condition that is characterized by short stature, intellectual disability, facial dysmorphology, and often retinal disorders [38]. The non-ocular features of Rubinstein-Taybi syndrome were not observed in the proband. A study using conditional KO *Ep300* mice in differentiating rods or cones using opsin driven Cre-recombinase showed that p300 is required for photoreceptor-specific chromatin organization, function, gene expression, and to maintain cell identity [39]. In addition, a recent study in zebrafish light-induced larvae suggested the role of EP300 in protecting photoreceptor cells from light-induced damage and shows that EP300 activation as a potential approach for the treating retinal degenerative diseases [40]. Taken together, this evidence suggests that *EP300* is as plausible candidate gene that is associated with retinal phenotype in this family. Loss of function may lead to a severe phenotype, while missense variant in this family may still retain all the essential domain of p300, thus only causes retinal phenotype.

Homozygosity mapping in combination with WES analysis detected a homozygous missense c.930C>G (p.(Phe310Leu) variant in Farnesyl-Diphosphate Farnesyltransferase 1 (*FDFT1*) in two siblings from family C with rod-cone dystrophy. *FDFT1* resides in the single homozygous region shared by the two affected siblings spanning 11.6 Mb. The variant affects a highly conserved phenylalanine, and a poorly conserved nucleotide (PhyloP: 1.09). The mutant leucine is also present in Baker’s yeast (Supplemental Figure S1). This substitution occurs in 87 out of 121,400 alleles (0.00072). The *FDFT1* gene encodes farnesyl-diphosphate farnesyltransferase 1 enzyme, which catalyzes the dimerization of farnesyl-diphosphate to form squalene in the mevalonate pathway. Two IRD-associated genes have previously been linked to the process of prenylation [41]. The mevalonate kinase (*MVK*) gene has been implicated in non-syndromic RP [42], and mutations in Member RAS Oncogene Family 28 (*RAB28*), encoding a prenylated RAB28 protein, are a cause of CRD [17]. Despite this ‘guilt by association’, the information provided above suggest this variant to be benign.

In family E with achromatopsia, we identified a homozygous c.769C>T (p.(Tyr257His)) substitution in the NDRG Family Member 4 (*NDRG4*) gene. This gene resides in the largest overlapping homozygous region of 76.5 Mb shared by the two affected siblings, but not by the healthy sibling. This highly conserved nucleotide variant (PhyloP: 4.48) is absent in the ExAC database and alters a highly conserved tyrosine. The variant is predicted to be damaging by all prediction programs and occurs in the Ndr domain of the NDRG4. The gene is expressed in the retina based on the ocular tissue database, human proteome map, Gepis tissue and human retinal transcriptome. No association or interaction with retinal-associated protein is known for this protein. When compared to the missense variant identified in this study, the *Ndrg4*-deficient mice present with impaired spatial learning and memory, but normal motor function [43]. Previous studies have shown that *Ndrg4* mRNA is expressed in the amacrine cells, horizontal cells, and ganglion cells in the developing mouse retina, and amacrine and possibly ganglion cells at 96 hours post fertilization in zebrafish [44].

A hemizygous c.164G>T (p.(Ser55Ile)) variant in Synaptotagmin Like 4 (*SYTL4*) was found in family H, which was not present in the ExAC database. No other family members were available to investigate the X-linked mode of inheritance. The substitution alters an evolutionary highly conserved serine, and also affects a highly conserved nucleotide with a PhyloP score of 5.21. The variant occurs in exon 4, which encodes the Rab binding domain. All the in silico tools suggest that the variant damages the protein. The larger size and the altered hydrophobicity of the new residue may disrupt the forming of the hydrogen bond between serine at position 55 with lysine at position 49 in the wild type situation. The altered residue is located on the protein surface, and thus may affect interaction with other molecules or protein domains. *SYTL4* encodes a member of the synaptotagmin like protein family, which contains the N-terminal Rab27 binding domain and C-terminal tandem C2 domain. The encoded protein binds to Syntaxin Binding Protein 1 (STXBP1), Member RAS Oncogene Family 3A (RAB3A), Member RAS Oncogene Family 8A (RAB8A) and Member RAS Oncogene Family 27B (RAB27B), and interacts with Myosin VA (MYO5A).

In family I, we identified a homozygous nonsense c.418C>T (p.(Arg140*)) variant in Transmembrane P24 Trafficking Protein 7 (*TMED7*). This variant is absent from the ExAC database. TMED7 might play an important role in vesicular protein trafficking. This variant will result in either a truncated protein product lacking the EMP24_GP25L or GOLD domain, or may be subjected to nonsense mediated decay.

Thus far, no disorder has been associated with mutations in this gene. Individuals carrying this variant demonstrated an early onset visual impairment in the first decade of life. In addition, nystagmus, developmental delay and tonic clonic seizures also occurred in the patient.

### 3.4. Score 1 Candidate Gene

We identified a homozygous missense variant c.4G>A (p.(Ala2Thr)) in G Protein-Coupled Receptor 45 (*GPR45*). This variant occurs in a homozygous state in two affected sisters with CRD in a consanguineous Israeli Muslim Arab family D. The variant is predicted to result in a small physicochemical change with a Grantham score of 58; the mutant threonine is larger than alanine, which may destabilize GPR45. The variant affects a moderately conserved nucleotide with a PhyloP score of 1.90 and the alanine at position 2 is moderately conserved up to frog. This variant is not present in the ExAC database and all in silico tools predict the variant to be deleterious. In addition, the variant resides outside the region that encodes functional protein domain. GPR45 is a member of the G protein-coupled receptor (GPCR) superfamily that is known to modulate physiological processes in the neurological systems [45].

## 4. Discussion

Whole exome sequencing has been instrumental for IRD gene discoveries since 2011 [15,46,47]. The implementation of this technology has contributed to the identification of ~40 novel genes that are associated with syndromic and non-syndromic IRDs in the last six years. Furthermore, novel genotype-phenotype correlations were identified for several IRD-associated genes by using exome sequencing. A tendency can be observed, as the early-discovered genes are more frequently mutated than the more recently identified genes. Previous genetic studies employing homozygosity mapping and WES in the Saudi Arabian population shows a small proportion of IRD cases (3–4%) that carry a mutation in novel IRD-associated genes [8,9]. Additionally, a gene panel study in 537 IRD cases demonstrated 79% of diagnostic yield in the genes that were discovered between 1995 and 2004 [48]. Together with challenges in interpreting novel variants, this low mutational load phenomenon led to the identification of many variants in novel candidate genes in singleton cases without any clear functional consequences.

The ERDC aims to identify the remaining unidentified IRD-associated genes by data sharing. In the past few years, the ERDC was able to identify several genes associated with IRDs [14,15,16,49]. To enable discovery of additional families harboring overlapping pathogenic variants the online Web-tool GeneMatcher may also be utilized [50]. This tool allows for researchers and clinicians to upload a gene with information about the identified genetic variants and the phenotype found in the patient that carries this variant or combination of variants.

Upon screening of WES and identity-by-descent data, 11 genes remained as candidates that occur in single families or cases. Using our pathogenicity assessment *SNRNP200*, *DHX32*, and *ZNF513* are the top ranked candidate genes based on the allele frequency, in silico prediction and interaction with the known IRD genes. Screening of IBD and WES data of unsolved IRD cases did not yield additional cases. The lower ranked candidate genes have generally weaker pathogenicity prediction scores, however all stand out when compared to other variants seen in the corresponding patients.

Besides the identification of novel candidate genes, we also revealed a novel mode of inheritance in two previously described IRD-associated genes. The *SNRNP200* variant is located in the Sec63 domain and predicted to be deleterious. Interestingly, an overlapping variant in this position, c.3269G>T (p.(Arg1090Leu), Figure 4C), has been previously identified in a large Chinese family segregating an autosomal dominant form of RP. *SNRNP200* encodes BRR2, a member of the spliceosome complex. Functional assays in yeast using a Brr2p construct containing p.(Arg1090Leu) demonstrated a splice defect, suggesting the association of *SNRNP200* with the disease phenotype. In the autosomal dominant RP associated variant, the wild type arginine is altered to a nonpolar and hydrophobic leucine. On the other hand, the autosomal recessive RP-associated variant alters to glutamine that is uncharged but has the same polarity as the wild type amino acid. The comparison of the two new residues in the 3D structure of SNRNP200 shows that the alteration to leucine shows a greater structural difference in comparison to glutamine. We hypothesize that p.(Arg1090Gln) only causes disease in a homozygous state, as its effect on the SNRNP200 activity is approximately half of the effect of p.(Arg1090Leu). The causality of autosomal recessive variants in *SNRNP200* was suggested by Wang et al. in a Chinese Leber congenital amaurosis patient who carried a homozygous p.(Pro1045Thr) variant [51]. Moreover, Bujakowska and colleagues proposed autosomal recessive inheritance in a RP patient with compound heterozygous variants in *SNRNP200* consisting of the same hypomorphic missense variant p.(Pro1045Thr) and a 1.1-Mb deletion spanning *SNRNP200* [52].

The *ZNF513* mutant residue Cys242 is predicted to have a larger size than the normal Arg242, which may destabilize the protein structure. The hydrophobicity of the wild type and mutant residue differs. Hydrophobic interactions, either in the core of the protein or on the surface, will be lost. The homozygous c.1015T>C (p.(Cys339Arg)) variant that was previously associated with autosomal recessive RP in a consanguineous family from Pakistan [53] is present three times in a homozygous state in ~60,000 ‘healthy’ individuals in ExAC, which sheds doubt on its pathogenic character. Knockdown of *znf513* in zebrafish model however suggested its role in retinal development and photoreceptor maintenance, as shown by the eye dysmorphology and photoreceptors loss in the knockdown model [53]. Possibly, the p.(Cys339Arg) variant shows reduced penetrance or is associated with variable age of onset of disease.

Among the score 2 classified genes, we identified a homozygous truncating variant in *TMED7*. The role of TMED7 in protein trafficking and lack of an associated disease marks *TMED7* as a putative target for further investigation. Similar to the other candidate genes, additional cases with variants in this gene would support its involvement in IRD.

In conclusion, we have identified 11 candidate IRD-associated genes through data sharing. To prove that these candidate genes are true IRD causative genes, whole genome sequencing in these cases could further confirm their candidacy by exclusion of other genetic causes. Moreover, additional unrelated cases need to be identified and additional functional evidence can support causality. For this, we encourage a worldwide genotype-phenotype data sharing in order to boost the completion of the IRD genetic puzzle.

## Figures and Tables

**Table 1:** Assessment of sequence variants identified in this study.

Gene name	Mode of Inheritance	Zygosity	DNA Variant	Protein Variant	CADD_Phred	ExAC ^$^ AC/AN/HA/AF	Described Protein Interaction	Known IRD-Associated Gene	Score *	Interpretation According to ACMG Guidelines #	
										Type of Evidence	Classification
*SNRNP200*	AR	Hom	c.3269G>A	p.(Arg1090Gln)	29.6	3/121,400/0/0.00002471	PRPF4; PRPF6; PRPF8 [27]	yes	4	PM1, PM5, PP1, PP2, PP3, PP4	LP
*DHX32*	AR	Hom	c.2176del	p.(Glu726Asnfs*57)	NA	2/121,276/0/0.00001649	FAM161A [31]	no	3	PVS1, PM4	P
*ZNF513*	AD	Het	c.724C>T	p.(Arg242Cys)	17.61	0/121,412/0/0	OPN1SW; PDE6A [27]	yes	3	PP2, PM2	US
*DSCAML1*	AR	Hom	c.925G>A	p.(Ala309Thr)	34	2/91,000/0/0.00002198		no	2	PP3	P
*EP300*	AD	Het	c.1069G>A	p.(Ala357Thr)	36	0/121,412/0/0		no	2	PP1, PP3, PM2	US
*FDFT1*	AR	Hom	c.930C>G	p.(Phe310Leu)	19.38	0/121,412/0/0		no	2	PP1, PP3, PM2	US
*NDRG4*	AR	Hom	c.769T>C	p.(Tyr257His)	27.4	0/121,412/0/0		no	2	PP1, PP3, PM2	US
*OTOGL*	AR	Het	c.2318C>T	p.(Pro773Leu)	27.6	89/118,882/2/0.0007486		no	2	PM3, PP3, BS2	US
*OTOGL*	AR	Het	c.2833C>T	p.(Arg945*)	NA	9/118,122/0/0.00007619		no	2	PVS1, PM3	P
*SYTL4*	XL	Hem	c.164G>T	p.(Ser55Ile)	26	0/121,412/0/0	RAB8A; RAB27A [32]	no	2	PP3, PM2	US
*TMED7*	AR	Hom	c.418C>T	p.(Arg140*)	37	0/121,412/0/0		no	2	PP1, PP3, PVS1, PM2	P
*GPR45*	AR	Hom	c.4G>A	p.(Ala2Thr)	13.47	0/121,412/0/0		no	1	PP1, PM2	US

*: Pathogenicity scoring based on four parameters: (a) In silico prediction; (b) Minor allele frequency; (c) Known interaction with retinal-associated protein; (d) Whether association with Inherited Retinal Disease (IRD) is known or not; Interpretation according to the American College of Medical Genetics (ACMG) guidelines #: Variant classification based on the ACMG guidelines; ExAC $: Variant absent from Exome Aggregation Consortium (ExAC) were indicated as zero alleles from total of 121,412 alleles included in ExAC; AC: Allele count; AD: autosomal dominant; AF: allele frequency; AN: allele number; AR: autosomal recessive; B: benign; BS: benign strong; HA: number of homozygous alleles; Hem: hemizygous; Het: heterozygous; Hom: homozygous; LP: likely pathogenic; NA: not analyzed; PM: pathogenic moderate; P: pathogenic; PP: pathogenic supporting; PS: pathogenic strong; PVS: pathogenic very strong; US: uncertain significance; XL: X-Linked.

**Table 2:** Clinical details of patients with mutations in candidate genes identified in this study.

ID/Gender /Origin	Age at Diagnosis/Age Recent Examination (yrs)	History	Visual Acuity (LogMAR) RE/LE	Refraction	Ophthalmoscopy	Full Field Electroretinogram	Goldmann Perimetry	Optical Coherence Tomography	Fundus Autofluorescence	**Other Symptoms**
**A-II:1, ♂** **Ghana**	42/58	Impaired visual acuity and night blindness in 5th decade	CF/CF	RE:S−1.00 C1.25 × 88°/ LE: S−1.25 C1.00 × 89°	Pale optic disks, mild attenuated retinal vessels, extensive atrophy of the RPE in the macula and midperiphery with hyperpigmentations, peripheral RPE spared	NA	Constricted up to 5°	Atrophy of the outer segments	NA	Lens sclerosis, Alpha-thalassemia
**B-II:1, ♀** **The Netherlands**	38/69	Night blindness in 3rd decade and visual field loss in the 4th decade	0.15/0.7	RE: S−1.75 C1.25 × 10°/ LE: S+0.50 C1.25 × 0°	Pale optic disks, severely attenuated retinal vessels, RPE alterations in the posterior pole, midperipheral RPE atrophy and peripheral bonespicule-like pigmentations	Photopic: severely reduced response, Scotopic: no reponse	Constricted up to 10°	Central and peripheral atrophy of the outer segments, intraretinal cysts	Hyper-autofluorescent macula with hypo-autofluorescent perifoveal ring and hypo-autofluoresent, patchy dots	Pseudophakia. RE traumatic corneal perforation and partial aniridia
**C-II:2, ♀** **Pakistan**	20-30/45	Adult onset nyctalopia and reduced vision, followed by loss of acuity	0.2/0.3	Emmetropic	White dots and intra-retinal pigment migration in mid-peripheral retina, especially above and below the optic disc. Bulls eye maculopathy	Rod-cone dysfunction	confrontation approximately 10°	Outer retinal degeneration, relative sparing of fovea	Concentric rings of hypo- and hyper-autofluoresence in the macula, consistent with a bulls eye maculopathy, and areas of hypo-autofluorescence in the mid-periperal retina	-
**D-II:1, ♀** **Muslim Arab**	Congenital/37	Congenital photophobia and reduced visual acuity, congenital nystagmus	0.1/CF 1m	NA	NA	Photopic: severely reduced responses, Scotopic: slightly reduced responses	NA	NA	NA	-
**D-II:2, ♀** **Muslim Arab**	Congenital/25	Congenital photophobia and reduced visual acuity, congenital nystagmus	CF 2m/CF 2m	NA	NA	Photopic: severely reduced responses, Scotopic: slightly reduced responses	NA	NA	NA	-
**E-II:3, ♀** **Turkey**	early infancy/4	Pendular nystagmus, reduced vision	1/4 APK/ 1/3 APK	RE: S+4.75 C−2.50 × 2°/LE: S+5.0 C−2.5 × 4°	no visible abnormalities	Photopic: reduced responses, Scotopic: NA	NA	NA	NA	-
**F-II:2, ♂** **Algeria**	2/22	Night blindness at 2 yrs, at 12 yrs minimal peripheral visual field, moderate photophobia	1.0/1.0	RE: S−0.50 C−0.50 × 55°)/LE: C−1.00 × 100°)	Depigmented peripheral retina with bone spicule pigmentary deposits in mid periphery. Macular reflex is normal. Narrowed retinal vessels and pale optic discs LE: interpapillomacular region is depigmented.	NA	Visual field is tubular, with the peripheral isopter V4e shortened at 15-20° around fixative point	CME with foveal thickness of 280 µm RE and 220 µm LE. Outer retinal layers are absent outside the fovea	Typical hypoautofluorescent spots in mid periphery towards the macula. A narrowed ring of hyperautofluorescence around the fovea. LE: hypoautofluorescent spots superonasal of the macula	-
**G-III:4, ♂** **Pakistan**	early infancy/15	Night blindness and visual field loss	1.0/1.0	NA	Attenuated retinal vessels, peripheral bone spicule pigmentation	NA	NA	NA	NA	-
**H-II:1, ♂** **The Netherlands**	16/37	Impaired visual acuity and visual field loss in the 2nd decade	NLP/HM 3m	RE: NA / LE: S−3.00 C1.75 × 177°	RE: phthisis bulbi, LE: Pale optic disk, attenuated retinal vessels, small island with remaining RPE in the macula, extensive atrophy and bone spicule pigmentations in the periphery	Photopic: moderately reduced response, Scotopic: severely reduced responses	LE: Peripheral island inferior	Disorganization and extensive atrophy of the outer retinal layers and thickened RPE in the fovea	NA	RE: Traumatic retinal detachments, aphakia. Proliferative vitreoretinopathy
**I-IV:2, ♀** **Asia**	1st decade/10	Reduced visual acuity, nystagmus	0.1/0.1	NA	Bull’s eye maculopathy	NA	NA	NA	NA	Developmental delay, tonic clonic seizures
**I-V:1, ♂** **Asia**	1st decade/12	Reduced visual acuity, nystagmus	0.2/0.3	NA	Bull’s eye maculopathy	NA	NA	NA	NA	Developmental delay, tonic clonic seizures
**J-IV:3, ♂** **France**	5-20/63	Night blindness and visual field loss	LP to 1.0/ LP to 1.0	Variable	Pigment deposits	No or reduced responses	Slightly decreased V4e isopter in periphery to tubular visual field	Moderate decrease of the outer nuclear layer in periphery to complete absence of outer nuclear layer	Typical hypoautofluorescence spots in retinal periphery	-
**K-IV:1, ♀** **The Netherlands**	41/53	Impaired visual acuity, and night blindness in the 5th decade	0.7/0.6	RE: S+2.50 C0.75 × 172° /LE: S+3.00 C1.50 × 165°	Mild pallor of the optic disk, mild attenuated retinal vessels, cystoid maculopathy, thinning of the RPE in the periphery with intraretinal bone spicule pigmentations (LE > RE)	Photopic: severely reduced responses, Scotopic: severely reduced responses	Peripheral sensitivity loss, no absolute defects	CME, peripheral atrophy of the outer segments	Hypoautofluorescent flecks in the fovea, hyperautofluorescent ring along the macula superimposed to the underlying edema, coarse hypoautofluorescent spots along the vascular arcades	Cystoid maculopathy, refractory to systemic treatment with Diamox^®^ and Octreotide^®^

APK: Amsterdam Picture Chart; BE: Both eyes; C: Cylindrical, CF: Counting fingers; CME: Cystoid macular edema; HM: Hand movement; LE: Left eye; LP: Light perception; NA: Not analyzed; NLP: Near light perception; m: Months; RE: Right eye; RP: Retinitis pigmentosa; S: Sphere, ♀: Female; ♂: Male.

## Supplementary Materials

The following are available online at www.mdpi.com/2073-4425/9/1/21/s1. Table S1: Provided details on whole exome sequencing and linkage/homozygosity approach. Figure S1: Amino acid conservation for the missense variants identified in FDFT1 in Human and Baker’s yeast.
